# Definition and diagnosis of Parkinson’s disease: guideline “Parkinson’s disease” of the German Society of Neurology

**DOI:** 10.1007/s00415-024-12687-5

**Published:** 2024-09-19

**Authors:** Franziska Hopfner, Günter Höglinger, German Parkinson’s Guidelines Group, Franziska Hopfner, Günter Höglinger, Mathias Bähr, Jos Becktepe, Daniela Berg, Kathrin Brockmann, Andrés Ceballos-Baumann, Joseph Claßen, Cornelius Deuschl, Günther Deuschl, Richard Dodel, Georg Ebersbach, Carsten Eggers, Thilo van Eimeren, Alessandra Fanciulli, Bruno Fimm, Ann-Kristin Folkerts, Madeleine Gausepohl, Alkomiet Hasan, Wiebke Hermann, Rüdiger Hilker-Roggendorf, Matthias Höllerhage, Wolfgang Jost, Elke Kalbe, Jan Kassubek, Stephan Klebe, Christine Klein, Martin Klietz, Thomas Köglsperger, Andrea Kühn, Paul Krack, Florian Krismer, Gregor Kuhlenbäumer, Johannes Levin, Inga Liepelt-Scarfone, Paul Lingor, Kai Loewenbrück, Matthias Löhle, Sylvia Maaß, Walter Maetzler, Regina Menzel, Philipp T Meyer, Brit Mollenhauer, Manuela Neumann, Per Odin, Tiago Outeiro, Monika Pötter-Nerger, René Reese, Kathrin Reetz, Olaf Rieß, Viktoria Ruf, Anja Schneider, Christoph Schrader, Alfons Schnitzler, Klaus Seppi, Friederike Sixel-Döring, Alexander Storch, Lars Tönges, Uwe Walter, Tobias Wächter, Tobias Warnecke, Florian Wegner, Christian Winkler, Karsten Witt, Dirk Woitalla, Kirsten Zeuner, Claudia Trenkwalder

**Affiliations:** 1 Department of Neurology with Friedrich Baur Institute, LMU University Hospital, Ludwig-Maximilians-University (LMU) Munich, Marchioninistr. 15, 81377 Munich, Germany; 2 German Center for Neurodegenerative Diseases (DZNE), Munich, Germany; 3 Munich Cluster for Systems Neurology (SyNergy), Munich, Germany; 4 Fachklinik Paracelsus-Elena-Klinik, Kassel, Germany; 5 Department of Neurosurgery, University Medical Center, Göttingen, Germany

**Keywords:** Parkinson’s disease, Definition, Diagnosis, Imaging, Genetics, Biomarkers

## Abstract

**Background:**

Accurate definition and operational criteria for diagnosing Parkinson’s disease (PD) are crucial for evidence-based, patient-centered care.

**Objective:**

To offer evidence-based recommendations for defining and diagnosing PD, incorporating contemporary clinical, imaging, biomarker, and genetic insights.

**Methods:**

The guideline development began with the steering committee establishing key PICO (patient, intervention, comparison, outcome) questions, which were refined by the coauthors. Systematic literature searches identified relevant studies, reviews, and meta-analyses. Recommendations were drafted, evaluated, optimized, and voted upon by the German Parkinson’s Guideline Group.

**Results:**

Parkinson’s disease (PD) is now understood to encompass a broader spectrum of etiologies than previously recognized. Advances in molecular pathogenesis, neuroimaging, and early clinical phenotypes suggest that PD is not a uniform disease entity and is often not idiopathic. This necessitates an updated framework for PD definition and diagnosis. The German Society for Neurology now endorses a broader concept of PD, incorporating both idiopathic and hereditary forms, as opposed to the previously narrower concept of “idiopathic Parkinson syndrome.” The revised guidelines recommend using the 2015 Movement Disorders Society diagnostic criteria, emphasize the importance of long-term clinical follow-up for improved diagnostic accuracy, and highlight the significance of non-motor symptoms in clinical diagnosis. Specific recommendations are provided for the use of imaging and fluid biomarkers and genetic testing to support the clinical diagnosis.

**Conclusion:**

The updated guidelines from the German Society for Neurology enhance diagnostic accuracy for PD, promoting optimized clinical care.

## Introduction

*Parkinson’s disease—origin and development of the disease concept:* Parkinson’s disease (PD) is named after James Parkinson, an English physician who first described the condition in 1817 in his essay titled “An Essay on the Shaking Palsy” [1]. In this work, Parkinson provided a detailed clinical description of what he termed “paralysis agitans” or the shaking palsy, which is the basis of the current concept of PD.

Parkinson’s essay was a seminal contribution to medical literature, as it was one of the earliest attempts to systematically describe a neurological disorder. James Parkinson observed several characteristic symptoms, including tremors, rigidity, and difficulty with movement, which are now recognized as hallmark features of PD. Parkinson’s astute observations laid the foundation for further research into the understanding and treatment of this condition.

The history of PD is marked by significant milestones, including the discovery of dopamine deficiency in the brains of affected individuals in the 1960s, which led to the development of dopamine-based therapies such as levodopa, a breakthrough medication that remains a cornerstone of Parkinson’s treatment. In addition, advancements in neuroimaging techniques, genetic studies, and experimental models have furthered our understanding of the disease pathology and paved the way for novel therapeutic approaches.

In recent years, the understanding of the disease has evolved significantly. Substantial progress has been made in elucidating the underlying pathogenic mechanisms, risk factors, and treatment options for PD. Currently, PD is believed to involve a combination of genetic, environmental, and neurodegenerative factors.

The terms “PD” and “idiopathic Parkinson’s syndrome” (IPS) were often used synonymously in the past. In recent years, however, it has become clear that a non-negligible number of patients with PD are affected by genetic variants and are, therefore, not “idiopathic” in nature. Therefore, the guideline commission of the German Society for Neurology recommends the use of the more general term PD.

This article summarizes the chapters focusing the definition and diagnosis of PD as approved by current standards in the national guidelines of PD by the German Society for Neurology.

## Methodology

Key PICO (patient, intervention, comparison, outcome) questions for the chapters were initially established by the steering committee of the Guideline Group “Parkinson’s Disease” commissioned by the German Society of Neurology and then refined by the respective chapter author groups. A systematic computer-based literature search based on citations collected by the National Library of Medicine—National Institutes of Health, was conducted based on these questions, identifying relevant studies, reviews, and meta-analyses. This identified literature was further supplemented by additional sources found by the chapter authors. The chapter authors drafted background texts and recommendations, which were then put to an online vote by all members of the German Parkinson Guideline Group. Recommendations that received less than 85% consensus were discussed in online meetings of the group. Recommendations with over 95% approval were considered to have “strong consensus,” while those with 75–95% approval were labeled as “consensus.”

The complete guideline was published in November 2023 by the DGN (www.dgn.org) and the Association of Scientific Medical Societies in Germany (AWMF, https://register.awmf.org/de/leitlinien/detail/030-010). This article presents an abbreviated and translated version of the guideline chapters addressing the definition and diagnosis of PD. A detailed description of the methodological approach can be found in the original guideline (in German) at: https://dgn.org/leitlinie/parkinson-krankheit.

### Nosology and Parkinson’s disease entities

A Parkinson syndrome is clinically defined by the presence of bradykinesia as essential symptom plus one or more of the features rigidity, tremor, or postural instability [2].

*Bradykinesia* is defined as slowing down in the initiation and execution of spontaneous and voluntary movements, assessed by finger tapping, hand movements, pronation–supination movements, toe tapping, and foot tapping [3]. In PD, bradykinesia is typically associated with a decrease in amplitude or velocity of continuous movements [3], whereas in atypical Parkinson syndromes, such as PSP, the decrease may be absent.

*Rigidity* is defined as velocity-independent resistance observed during passive movements in large joints. A cogwheel phenomenon may be present. A cogwheel phenomenon without resistance is not considered rigidity [4].

*Tremo****r*** is defined as an involuntary rhythmic movement of one or more body parts. Rest tremor, defined as a 4–6 Hz tremor occurring in completely relaxed limbs and suppressed during movement, is characteristic but not obligatory for the diagnosis of Parkinson’s disease [3]. Further forms of tremor (and in very rare cases also rest tremor) may also occur in atypical Parkinson syndromes.

*Postural instability* is defined as a tendency to fall not caused by primary visual, vestibular, cerebellar, or proprioceptive dysfunction [5]. While it is frequently and early observed in atypical Parkinson syndromes, postural instability occurs late in PD and is, therefore, no longer considered a cardinal motor symptom of PD according to current diagnostic criteria [3].

### Symptomatic (i.e., secondary) Parkinson syndrome

A Parkinson syndrome caused by an identifiable, non-genetic cause is referred to as symptomatic or secondary Parkinson syndrome, as exemplified in Table 1.

**Table 1:** Main causes of symptomatic Parkinson syndromes

Classification	Causes (examples)
Hydrocephalus	E.g., normal pressure hydrocephalus
Drug-induced Parkinson syndrome	E.g., antidopaminergics, metoclopramide, and many others
Metabolic causes	E.g., Wilson’s disease
Cerebrovascular lesions	E.g., strategic infarctions or chronic vascular encephalopathy
Neoplastic lesions	E.g., midline-associated tumors
Encephalitic/post-encephalitic lesions	E.g., HIV encephalitis, encephalitis lethargica
Toxin-induced lesions	E.g., poisoning with carbon monoxide, carbon disulfide, cyanide, manganese, methanol, MPTP, MDMA
Traumatically induced lesions	E.g., chronic traumatic encephalopathy or acute shear injuries

*Cave:* Gait disturbance observed in normal pressure hydrocephalus or chronic vascular encephalopathy is often referred to as lower body parkinsonism. However, in these conditions, gait apraxia may sometimes occur, caused by frontal lobe dysfunction. Therefore, these are considered more as a differential diagnosis rather than Parkinson syndrome.

To facilitate comprehension, all terms pertaining to the definition of the illness are succinctly presented in Table 2.

**Table 2:** Lexicon of the recommended nomenclature

Term	Description	References
Symptomatic (i.e., secondary) Parkinson syndrome	A Parkinson syndrome caused by an identifiable, non-genetic cause is referred to as symptomatic or secondary Parkinson syndrome	[6]
Hereditary Parkinson’s disease	Parkinson’s syndrome, which is caused by rare or rare or less frequent pathogenic genetic variants (“mutations”); with regard to the nomenclature for hereditary Parkinson’s syndromes, we propose to follow the recommendations of the International Parkinson and Movement Disorder Society Task Force (e.g., PARK-*SNCA*, and PARK-*GBA1*)	[6–8]
Idiopathic Parkinson’s syndrome (IPS)	Idiopathic Parkinson’s syndrome with unknown cause, i.e., not symptomatic, not hereditary (the term is, therefore, not strictly speaking identical to PD)	[6]
Parkinson’s disease (PD)	Lewy body disease, which leads to Parkinson’s syndrome (the term can be used be used in the presence of a clinically manifest Parkinson’s syndrome is clinically manifest, but also for conditions that future development of Parkinson’s disease syndrome, e.g., in the case of premotor or prodromal PD)	[9–11]
Preclinical Parkinson’s disease	Lewy body disease at a very early stage, which does not yet show obvious clinical symptoms	[12]
Premotor Parkinson’s disease	Lewy body disease in the early stages, which only shows non-motor symptoms, e.g., REM sleep behavior disorder	[12]
Prodromal Parkinson’s disease	Lewy body disease in the early stages, which presents with non-motor symptoms and mild-motor signs, but does not yet meet the criteria for the diagnosis of a classic Parkinson’s syndrome	[13, 14]
Parkinson’s disease with dementia (PDD)	Lewy body disease in the advanced stage, which is associated with a neurocognitive disorder, which manifests itself at the earliest 1 year after the onset of motor symptoms	[15]
Dementia with Lewy bodies (DLB)	Neurocognitive disorder caused by a Lewy body disease. Generic term for PD with dementia and Lewy body dementia	[15]
Lewy body disease (LBD)	Disease characterized by the presence of alpha-synuclein-immunoreactive Lewy bodies and Lewy neurites in certain cortical and cortical and subcortical regions of the brain. Generic term for PD, PD with dementia and dementia with Lewy body	[16, 17]
Atypical Parkinson’s syndrome (APS)	Parkinson’s syndrome, which is caused by a neurodegenerative disease other than Lewy disease (i.e., a generic term for diseases such as a generic term for diseases such as multisystem atrophy (MSA), corticobasal syndrome (CBD) and progressive supranuclear palsy (PSP))	[6, 18]

### Hereditary Parkinson’s disease

Hereditary PD is caused by rare pathogenic gene variants in single genes (commonly called monogenic mutations) [7, 8, 19]. Pathogenic variants in recessive genes (e.g., *PRKN*) have high penetrance, meaning that almost all variant carriers develop the disease [7, 20]; in carriers of pathogenic variants in dominant genes, penetrance is usually age-dependently reduced (e.g., *LRRK2*), since a significant portion of variant carriers remain healthy throughout life or develop the disease late [6–8]. Genes have been identified for both, classic PD, and atypical Parkinson syndromes or dystonias with Parkinson syndrome. This guideline focuses exclusively on genes associated with clinically classical Parkinson syndromes, allowing of the diagnostic label of hereditary PD. Regarding the nomenclature for hereditary PD, we suggest following the recommendations of the International Parkinson and Movement Disorder Society Task Force (www.mdsgene.org/g4d) (Table 2).

Sporadic PD is genetically complex, attributable to genetic variants in numerous genes each contributing weakly to the risk of disease (i.e., polygenic). Heterozygous mutations in the glucocerebrosidase gene (*GBA1, PARK-GBA1*) take a special position, since they cover the entire spectrum from weak to strong risk factors, depending on the specific variant [19]. Some highly pathogenic variants confer a high disease risk similar to weakly pathogenic variants in genes counted among the monogenic causes of Parkinson syndrome. In this guideline, *GBA1* is counted among the strong risk factors.

Non-inheritable forms of PD are referred to as “sporadic PD.” For sporadic Parkinson syndromes, a large number of common genetic variants (SNPs, single nucleotide polymorphisms) have been identified through genome-wide association studies, with each individual variant contributing only slightly to the risk of developing a Parkinson syndrome. The inheritance of individual risk variants does not follow Mendelian rules. Polygenic risk scores aggregate the risk of multiple single nucleotide polymorphisms and can explain a higher proportion of the risk. The steadily growing amount and availability of molecular biological data will lead to an increasingly better understanding of the genetic complexity of Parkinson syndromes and open up possibilities for additional subclassifications and ultimately personalized therapies.

### Idiopathic Parkinson syndrome

The terms “idiopathic Parkinson syndrome” (IPS) and “Parkinson’s disease (PD)” have often been used synonymously in the past. However, in recent years, it has become clear that some cases of PD are caused by genetic variants (e.g., heterozygous *GBA1* mutations, autosomal-dominant *LRRK2* mutations; details see www.mdsgene.org/g4d). Therefore, for patients without an identifiable cause, the term “idiopathic PD” is currently used in the international literature, while patients affected from rare or less common pathogenic genetic variants are referred to as “hereditary (familial) Parkinson syndromes,” even if heritability is not apparent in the family history (e.g., due to small families, de novo mutations, or reduced penetrance). With increasing knowledge and better understanding of the pathophysiological processes underlying Parkinson syndromes, the term “idiopathic” may become obsolete in the future (Table 2).

### Parkinson’s disease

The term PD can be used both in the presence of a clinically established Parkinson syndrome and in the context of specific symptom constellations that indicate the future development of a Parkinson syndrome, such as premotor or prodromal PD. In the vast majority of cases, PD is non-inheritable and referred to as “sporadic PD.” Sporadic PD is a Lewy body disease that results in a Parkinson syndrome. In rare cases, mutations (e.g., in the genes *SNCA*, *LRRK2*, and *GBA1*) can be identified, which are then classified as “hereditary Parkinson syndromes.” Parkinson syndromes associated with mutations in other genes (e.g., *VPS35*, *DJ1*, *PARKIN*, and *PINK1*) typically do not exhibit the typical histopathological features of Lewy body disease. Hereditary PD should be reported with the specific nomenclature recommended by the International Parkinson and Movement Disorder Society Task Force (e.g., PARK-*VPS35*) (Table 2).

*Preclinical PD* is defined as Lewy body disease in a very early stage that does not yet exhibit obvious motor or non-motor clinical signs (Table 2) [12].

*Premotor PD* is characterized by Lewy body disease in an early stage that, at the time of examination, only presents non-motor symptoms, such as REM sleep behavior disorder, olfactory dysfunction, and/or depression (Table 2) [12, 13].

*Prodromal PD* refers to Lewy body disease in an early stage that, at the time of examination, exhibits non-motor symptoms and only mild-motor symptoms but does not yet meet the criteria for the diagnosis of classical Parkinson syndrome (Table 2) [12, 13].

*PD with dementia (PDD)* is defined as advanced-stage Lewy body disease accompanied by a neurocognitive disorder occurring more than 1 year after the manifestation of motor symptoms of PD (Table 2) [9, 15].

*Dementia with Lewy bodies (DLB)* is defined as Lewy body disease primarily presenting as a neurocognitive disorder, with the onset preceding or following the appearance of motor symptoms of PD by less than 1 year. Neuropathological examination of DLB typically reveals higher stages of Alzheimer-associated changes (amyloid plaques and neurofibrillary tangles) compared to pure PDD (Table 2) [21].

The term *Lewy body dementia* refers to all types of dementia associated with Lewy body disease (i.e., an umbrella term for PDD and DLB) (Table 2).

### Lewy body disease

Lewy body disease (LBD) is an umbrella term for clinically heterogeneous neurodegenerative disorders (i.e., PD, including preclinical, prodromal, and premotor PD, PDD, and DLB), defined by alpha-synuclein (aSyn)-immunoreactive Lewy bodies and Lewy neurites in specific cortical and subcortical brain regions [22]. The gold standard for definitively diagnosing LBD currently involves (post-mortem) neuropathological examination of the central nervous system [23]. While specific characteristics are described for each of the various clinical entities of LBD (e.g., regarding regional distribution and severity of neurodegeneration and Lewy body pathology), given the fluid transitions between entities and considerable overlaps, assigning an individual case to one of these entities is only possible within a clinical–pathological context [24]. In some cases, the diagnosis of LBD can be made genetically by identifying a pathogenic rare or uncommon genetic variant in SNCA, LRRK2, or GBA1 that is clearly associated with LBD pathology (Table 2) [16, 17].

### Atypical Parkinson syndrome

Atypical Parkinson syndromes are defined as a group of Parkinson syndromes caused by a neurodegenerative disease other than Lewy body disease, i.e., an umbrella term for diseases such as multiple system atrophy (MSA), corticobasal degeneration (CBD), and progressive supranuclear palsy (PSP) (Table 2) [6, 18].

## Clinical diagnostic criteria

### Question 1: how effective is the diagnosis of Parkinson’s disease by a clinical movement disorder expert using the UK Parkinson’s Disease Society Brain Bank criteria compared to the post-mortem diagnosis?

#### Background

In medical care, PD must be accurately clinically differentiated from secondary Parkinsonian syndromes and other Parkinsonian syndromes with our without neurodegenerative diseases. A clinical diagnosis of PD should be based on recognized criteria. The UK Parkinson’s Disease Society Brain Bank criteria [2, 3] are still frequently applied in clinical and scientific practice.

#### Results

Three studies validating clinically diagnosed cases using the UK Parkinson’s Disease Society Brain Bank criteria against histopathological post-mortem results in Parkinsonian syndromes at different disease stages have been published [3, 4, 25]. These studies suggest that the clinical diagnosis of PD can be made using the UK Parkinson’s Disease Society Brain Bank criteria [2].

#### Recommendation (new in German guideline, 2023)

It may be considered to diagnose PD using the UK Parkinson’s Disease Society Brain Bank criteria. However, the criteria have limitations in terms of diagnostic certainty, since they include the clinical course as requirement for the diagnosis, and other criteria (see below) are available as alternatives.

*Consensus strength*: 97.4%, strong consensus.

### Question 2: how effective is the diagnosis of Parkinson’s disease by a clinical movement disorder expert using the UK Parkinson’s Disease Society Brain Bank criteria compared to long-term clinical follow-up?

#### Background

The diagnostic criteria for PD primarily rely on the UK Parkinson’s Disease Society Brain Bank criteria, the only neuropathologically well-validated criteria so far. Key diagnostic criteria include bradykinesia, resting tremor, asymmetrical symptom presentation, and disease progression. Supportive diagnostic parameters include levodopa-responsiveness, long-term observation of at least 5 years, motor fluctuations, and dyskinesias.

#### Results

In a longitudinal study from Arizona, 232 patients were included, of whom 131 had possible or probable PD. They were evaluated annually with UPDRS III and medication records [26]. Patients showing good response to levodopa, at least two classic clinical signs (e.g., tremor and bradykinesia), and no symptomatic causes were classified as probable PD. After their death, neuropathological examination confirmed the diagnosis in 89 patients and ruled it out in 42. Long-term observation was crucial: patients with classic symptoms and a positive response to levodopa but disease duration of less than 5 years had a 53% positive predictive value, increasing to 88% with more than 5 years’ duration. The presence of motor fluctuations or dyskinesias further raised the positive predictive value to 92% and 96%, respectively.

#### Recommendation (new in German guideline, 2023)

Observing the disease progression for more than 5 years improves the likelihood of making a correct diagnosis from 53% to > 85% and is, therefore, superior to solely applying the UK Parkinson’s Disease Society Brain Bank criteria, indicating that Parkinson’s patients should always be re-evaluated over a long-term course.

During this process, the occurrence of motor fluctuations and dyskinesias should be considered both by examination and patient history, as these symptoms significantly enhance the likelihood of making a correct diagnosis, thus representing an important clinical feature in the long-term course.

*Consensus strength*: 100%, strong consensus.

### Question 3: what are the specificity and sensitivity of the MDS criteria for the clinical diagnosis of Parkinson’s disease compared to the criteria of the UK Parkinson’s Disease Society Brain Bank?

#### Background

In 2015, the new and currently active criteria of the Movement Disorder Society (MDS criteria) for diagnosing PD have been introduced [9]. Supportive criteria include the levodopa test, levodopa-induced dyskinesias, unilateral rest tremor, non-motor symptoms, and additional instrumental examinations such as the DAT (dopamine transporter) Scan (with a normal result serving as an exclusion criterion) and metaiodobenzylguanidine (MIBG) scintigraphy.

#### Results

The MDS criteria of 2015 introduce several distinctions [9]. They categorize negative and positive features, where negative features include absolute exclusion criteria and red flags. Positive features include supportive criteria, which can mitigate the impact of red flags if present. Exceptions to absolute exclusion criteria accommodate special cases. Importantly, dementia is not an exclusion criterion, as it may manifest early as mild cognitive impairment (MCI) in PD and later progress to PDD clinically. Similarly, early cognitive impairments, fluctuations in vigilance, and hallucinations indicative of DLB are not exclusion criteria, as DLB is viewed as a continuum within the spectrum of PD manifestations [24]. The clinical progression over time remains of relevance for the criteria. For the first time, a non-motor symptom (hyposmia) is also considered a supportive criterion. Clinical motor criteria include cardinal features of a Parkinson’s syndrome such as bradykinesia, rigidity, and tremor. A positive response of > 30% on UPDRS III (motor assessment) after an acute levodopa test is considered a supportive criterion. Other supportive criteria include levodopa-induced dyskinesias, rest tremor in a limb, and positive findings from other diagnostic tests (such as olfactory testing or MIBG scintigraphy, indicating cardiac sympathetic denervation). A clinically established diagnosis (aiming for at least 90% specificity with slightly lower sensitivity) can be made if at least two supportive criteria are met without red flags or exclusion criteria. Criteria for a clinically probable diagnosis (targeting at least 80% specificity with at least 80% sensitivity) are met if one red flag is balanced by one supportive criterion, or two red flags by two supportive criteria. More than two red flags or any exclusion criteria should not be present. In a subsequent study by Postuma in 2018 [22], these new MDS criteria were validated against clinical experts’ diagnosis. This multicenter study involved clinical movement disorder experts and inexperienced neurologists assessing 434 patients with PD and 192 with Parkinsonian syndromes of other etiologies. Experts classified patients, while inexperienced neurologists used the newly developed MDS criteria for diagnosis. The overall diagnostic accuracy for probable PD was 92.6%, with an overall error rate of 7.4%. Specificity and sensitivity were higher using the MDS criteria (no neuropathological confirmation) compared to the previously standard UK Brain Bank criteria, which had an accuracy of 86.4% and an error rate of 13.6%, with specificity improving with disease duration.

#### Recommendation (new in German guideline, 2023)

For diagnosing PD, the MDS criteria from 2015 should be applied. The sensitivity and specificity of the MDS criteria surpass those of the UK Brain Bank criteria. Long-term disease management under the guidance of an expert improves diagnostic accuracy, necessitating regular follow-up examinations.

*Consensus strength*: 94.7%, consensus.

Important yet insufficiently answered research question: The MDS criteria for diagnosing PD should be validated against the neuropathological diagnostic gold standard.

### Question 4: how effective is the acute levodopa test or an apomorphine test compared to long-term clinical follow-up for diagnosing PD?

#### Background

Many PD patients show clinical improvement with a single dose of oral levodopa and/or subcutaneous apomorphine. The test sometimes comprises pre-treatment with domperidone for > 24 h to prevent side effects. A standardized dose of levodopa (150–250 mg) is administered orally after a pause of the patient’s anti-Parkinson medication for several hours (e.g., 12 h). Alternatively, apomorphine injection can be used (e.g., starting with stepwise doses such as 1.5 mg, then 3 mg, and 4.5 mg). The motor score of the Unified Parkinson’s Disease Rating Scale (MDS-UPDRS part III) is commonly used to measure the effect (before and approximately 1 h after levodopa administration or 15–20 min after apomorphine injection). A systematic review and diagnostic study examined the effectiveness of levodopa and apomorphine tests in diagnosing individuals with Parkinsonian syndromes [27].

#### Results

Merello et al. [28] conducted a blinded evaluation of the MDS-UPDRS, finding the sensitivity and specificity of the acute levodopa test with 250 mg levodopa/carbidopa to be 70.9% and 81.4%, respectively, with a positive predictive value of 88.6%.

A review encompassing 13 studies reported sensitivity ranging from 75 to 86% and specificity from 85 to 87% for diagnosing PD using apomorphine or levodopa tests [27]. It revealed no superiority of acute apomorphine or levodopa tests over chronic levodopa therapy for confirming a PD diagnosis. In fact, de novo PD patients responded better to chronic levodopa treatment.

Another study followed 134 PD patients over 3 years to assess the predictive value of levodopa and apomorphine tests regarding diagnosis and dopaminergic therapy effect [29]. The study included 83 patients diagnosed with PD based on clinical progression using UK Brain Bank criteria and/or autopsy, and 51 control patients with atypical or unclassified Parkinsonian syndromes. Patients received oral levodopa/carbidopa (250 mg) or subcutaneous apomorphine (1.5, 3, and 4.5 mg), and clinical effect was measured using repeated UPDRS III evaluations. Those showing at least a 16% improvement in UPDRS III after the acute levodopa or apomorphine test likely had PD, with sensitivity ranging from 70 to 77% and specificity from 63.9% to 71.1% for diagnosing PD using these tests. Overall, sensitivity and specificity were higher in the levodopa test compared to the apomorphine test [29]. In addition, patients who responded well to levodopa over 3 years were compared to those who did not. Predicting a positive chronic therapy response was most accurate when patients improved by at least 14.5% on UPDRS III in the levodopa test, with a sensitivity of 69.4% and specificity of 79.4% using this cutoff.

In another study of 210 PD patients followed up after 2 years, combining a positive levodopa test with hyposmia detected in a smell test (Sniffin’ Sticks) increased diagnostic sensitivity to 90% and specificity to 74% [30].

Potential drawbacks of levodopa and apomorphine tests include the need for domperidone pre-treatment (bearing the risk of prolonging the cardiac QT interval), risk of side effects, variability in test methodology and evaluation criteria, time and cost [27]. Common apomorphine test side effects include drowsiness, nausea, vomiting, hypotension, and sweating. Levodopa has fewer side effects than apomorphine, but nausea, vomiting, and orthostatic hypotension can occur.

#### Recommendation (new in German guideline, 2023)

Acute levodopa and apomorphine tests for diagnosing PD should not be performed routinely.

However, the acute levodopa test serves as a supportive criterion in the MDS Parkinson’s criteria when improvement is significant, with UPDRS III > 30%, and thus may be considered in the early diagnosis of PD.

*Consensus strength*: 100%, strong consensus.

### Question 5: how effective is the therapeutic response to levodopa therapy compared to long-term clinical follow-up for diagnosing PD?

#### Background

PD diagnosis relies on classic clinical criteria such as bradykinesia, resting tremor, and asymmetry of symptoms. It also includes several non-motor symptoms like hyposmia. Additional diagnostic parameters include responsiveness to levodopa, long-term observation, and the development of motor fluctuations and dyskinesias over time.

#### Results

In the study by Adler et al. [19], which investigated patients neuropathologically, patients were classified into two diagnostic categories: (1) probable Parkinson’s Disease (ProbPD): these patients exhibited two out of three cardinal signs, including resting tremor, bradykinesia, and rigidity. There were no indications of a symptomatic cause, but there was improvement with dopaminergic medication and a continuous therapeutic response to dopaminergic therapy.

(2) Possible Parkinson’s disease (PossPD): patients in this category also had two out of the three cardinal signs, a symptom duration ≤ 5 years, and either no testing of dopaminergic therapy or insufficient dosage. This group comprised 34 patients, of whom 31 had never been treated with levodopa and three had received inadequate dosages. Among these patients, only 9 had a neuropathologically confirmed diagnosis of PD, resulting in a positive predictive value of only 26%. However, the duration of disease was only 0.7 years in this subgroup. The authors noted that due to the small number of patients, it was not possible to assess improvements in clinical diagnostic accuracy based on specific clinical signs.

Once responsiveness to levodopa was demonstrated, patients transitioned from Group 2 (PossPD) to Group 1 (ProbPD). In these cases, a positive response to levodopa therapy improved the diagnostic accuracy from 26 to 53%.

For patients with classic symptoms and responsiveness to levodopa therapy, but a disease duration of < 5 years, the positive predictive value for diagnosing PD was 53%, which increased to 88% with a disease duration of > 5 years. Furthermore, when motor fluctuations or dyskinesias occurred, the positive predictive value improved further to 92% and 96%, respectively.

In summary, the positive predictive value of PD diagnosis can be significantly enhanced by long-term observation of the clinical course and surpasses the criterion of responsiveness to levodopa.

#### Recommendation (new in German guideline, 2023)

The therapeutic response to levodopa therapy can improve diagnostic accuracy. However, diagnostic accuracy is significantly higher with a longer duration of treatment ≥ 5 years, making long-term clinical follow-up superior to a diagnosis based solely on responsiveness to levodopa therapy for diagnosing PD.

Disease progression should be monitored for more than 5 years, including regular clinical assessments, to confirm PD diagnosis and guide appropriate therapeutic decisions.

*Consensus strength:* 100%, strong consensus.

### Question 6: how effective is the therapeutic response to dopamine agonist therapy compared to long-term clinical follow-up for diagnosing Parkinson’s disease (PD)?

#### Background

The differential diagnosis of different Parkinson syndromes often poses major challenges, especially in the early years of the disease. This leads to the search for definitive predictors for PD or atypical Parkinson syndromes. Clinical experience indicates that patients with PD typically experience substantial improvement in cardinal symptoms, except tremor, with a dopamine agonists. In contrast, most atypical Parkinson syndromes show limited or no symptom improvement with such treatments over time. This underpins the clinical practice of the treatment with a dopamine agonist: a clinical improvement of > 30% in UPDRS III is considered a “positive response,” indicative of PD, whereas < 30% improvement is termed “lack of response.” Patients with atypical Parkinson syndromes often initially respond positively to dopaminergic stimulation, albeit partially and temporarily.

#### Results

While the lack of response to a dopamine agonist is generally considered to support a diagnosis of PD, the scientific evidence for a strong recommendation is lacking due to the absence of systematic prospective long-term studies to date.

#### Recommendation (new in German guideline, 2023)

Due to lack of evidence, no recommendation can be made regarding the effectiveness of dopamine agonist therapy for predicting a correct diagnosis of PD.

*Consensus strength:* 97%, strong consensus.

### Question 7: is it beneficial to incorporate the MDS prodromal criteria in the early-stage diagnosis of PD compared to clinical follow-up over 3–5 years?

#### Background

The classic motor symptoms of bradykinesia, rigidity, and rest tremor, which enable the diagnosis of PD, are typically preceded by a phase lasting years to decades during which the underlying neurodegenerative process spreads in the nervous system, but has not yet reached the extent required in the relevant brain areas for the manifestation of these classic motor symptoms. However, during this so-called prodromal phase, the neurodegenerative process can lead to other non-motor symptoms or motor symptoms of milder severity that are typical but not specific to the underlying disease.

Based on these facts, the International Parkinson and Movement Disorders Society (MDS) has developed criteria using Bayesian statistics to calculate the probability that an individual, with certain prodromal symptoms and risk factors for PD (e.g., pesticide exposure and genetic predisposition), is in the prodromal phase of the disease or not. A criterion for including prodromal markers and risk factors in the statistical calculation was that at least two longitudinal prospective studies were available to calculate a “plausibility quotient” (likelihood ratio, LR). This LR, along with the individual’s age, is integrated into a probability formula that calculates the individual likelihood of the prodromal phase based on the presence or absence of certain symptoms. The prodromal criteria, first published in 2015, were updated in 2019 [13, 14].

#### Results

Prodromal criteria for PD, based on prospective longitudinal studies, effectively summarize risk factors and prodromal markers [13, 14]. Applying these criteria in the early stages of PD can support diagnosis. This applies to individual symptoms, such as the presence of REM sleep behavior disorder (RBD), as well as combinations of symptoms. Using the complete prodromal criteria in individuals where a diagnosis has not yet been established can lead to a diagnosis of “probable prodromal PD” or “possible PD” upon reaching certain probability thresholds.

In a prospective longitudinal study in the general population, individuals classified as having “probable prodromal PD” demonstrated a specificity for predicting PD after 3 years of 98.8% and a sensitivity of 66.7%, with a positive predictive value of 40.0% [31]. Over 5 and 10 years, specificity remained stable while sensitivity decreased, and the prospective predictive value increased. Although a direct comparison with a clinical course over 3–5 years is lacking, the presence of a “probable prodromal stage” can support the diagnosis of PD in its early years when motor symptoms are less pronounced. It is important to note that the full presentation of motor symptoms and response to dopaminergic therapy significantly contribute to diagnostic certainty in clinical PD diagnosis, independent of prodromal criteria.

#### Recommendation (new in German guideline, 2023)

The application of the MDS prodromal criteria in the early phase of PD is beneficial and should be considered in clinical practice to enhance diagnostic certainty in the initial years. Continuous monitoring and reassessment of the diagnosis are crucial, especially in the early stages of the disease.

*Consensus strength*: 97.1%, strong consensus.

### Question 8: what is the predictive value of validated olfactory testing for detecting hyposmia, as compared to clinical follow-up for the diagnosis of PD?

#### Background

Hyposmia is a non-motor symptom of PD that often manifests in the prodromal phase. Its presence at the onset of motor symptoms enhances diagnostic certainty. Olfactory impairments are frequently underreported and may not be consciously recognized by patients. Objective assessments of olfactory function can be conducted using bedside tests such as the University of Pennsylvania Smell Identification Test (UPSIT) or the Sniffin’ Sticks Test, commonly used in Germany [32]. These tests distinguish between different domains of smell (detection, identification, discrimination). The extent of neurodegeneration in the olfactory bulb and associated centers, and thus the presence of hyposmia, may vary depending on the individual course of the disease [33].

#### Result

Olfactory impairments can be assessed subjectively or objectively using various test methods. These impairments are often subjectively unnoticed (up to 72% of Parkinson’s patients) [32] or only reported upon explicit request, validating the utility of standardized bedside tests. Routine options include easily applicable tests such as the University of Pennsylvania Smell Identification Test (UPSIT), its derived short version B-SIT (Brief 12-item Smell Identification Test), and the Sniffin’ Sticks Test, with the latter being the most commonly used. UPSIT includes some odors not universally recognized, thus adjusted for various cultural contexts. B-SIT was designed to overcome these cross-cultural differences. Sniffin’ Sticks Test presents various odor-containing sticks, evaluated across 3 modalities (identification, threshold, discrimination). A study indicated that the Sniffin’ Sticks Test’s identification subscore performed nearly equivalently to its total score (including threshold, identification, discrimination), allowing for simplified testing procedures [32]. More complex olfactory dysfunction assessments like olfactory evoked potentials are limited to specialized centers. Olfactory impairments are common in the general population, affecting approximately 60–80% of individuals aged over 80 [34], hence isolated hyposmia initially viewed as nonspecific, potentially arising from conditions such as viral illnesses, smoking or nasal surgeries. Screening studies for idiopathic hyposmia have identified few patients who later developed PD [35]. Conversely, large-scale epidemiological studies and meta-analyses have shown a clear association between hyposmia and PD, often preceding motor symptoms [36, 37]. However, up to 44% of PD patients may present with normosmia during their disease course [38]. Baseline data from the Parkinson’s Progression Marker Initiative (PPMI), a multicenter observational study of early untreated Parkinson’s patients (*n* = 416), revealed hyposmia in only 34.9%. Current studies suggest olfactory dysfunction worsens with PD progression [39]. Patients with clinically isolated REM sleep behavior disorder (iRBD) in the prodromal stage are more likely to develop PD (conversion) when presenting with hyposmia compared to normosmia [16, 40]. This finding is supported by revised MDS prodromal criteria, indicating a likelihood ratio (LR) of 6.4 for PD development with motor symptoms and hyposmia [13]. In PD patients, hyposmia and REM sleep behavior disorder are associated with poorer prognosis, including worse motor and cognitive symptoms [41–43]. Based on a clinical–genetic classification model established in the PPMI cohort, incorporating age, gender, olfactory function (measured by UPSIT), genetic risk, and family history positively correlates with high sensitivity (0.834) and specificity (0.903) in discriminating PD from controls [44]. Applying this model to other cohorts (e.g., PARS) yielded even better AUC values in ROC analysis [44]. In differential diagnosis, a small cohort study comparing PD with atypical Parkinson syndromes (MSA, PSP, DLB) and other conditions (e.g., essential tremor) found B-SIT-diagnosed hyposmia distinguished PD from non-Parkinson syndromes (e.g., essential tremor), yet did not differentiate PD from atypical Parkinson syndromes nor differentiate atypical Parkinson syndromes from other conditions (like essential tremor) [45]. Differentiating all Parkinson syndromes (PD atypical Parkinson syndromes, vascular Parkinson’s syndrome) from other conditions (e.g., essential tremor) resulted in 80% sensitivity, 40% specificity, 80% positive predictive value, 40% negative predictive value, and 70% diagnostic accuracy. In a 2-year retrospective analysis, differential diagnosis of PD from other Parkinson syndromes, using standardized levodopa response alone or combined with hyposmia testing via Sniffin’ Sticks, improved diagnostic accuracy (accuracy) by increasing sensitivity (from 0.70 to 0.90) while decreasing specificity (0.90 to 0.74), maintaining nearly stable positive predictive value (0.97 to 0.91), and significantly increasing negative predictive value (from 0.52 to 0.72) [30]. However, clinical diagnosis served as the reference criterion in this study, precluding comparison between hyposmia testing and purely clinical progression. Conversely, the “Arizona Study of Aging and Neurodegenerative Disorders” (AZSAND, [34]) histopathologically confirmed PD diagnosis, validating clinical diagnosis further. In this study, diagnostic accuracy was lower with a disease course of less than 5 years and/or no response to medication. “Possible PD” and “probable PD” were predefined; histopathological confirmation was achieved in only 22% of patients with possible PD. While two out of three cardinal symptoms were present, disease duration was less than five years with most patients untreated or unresponsive to medication. In contrast, meeting “probable PD” criteria (2/3 cardinal symptoms, excluding symptomatic causes, positive response to medication) increased diagnostic confirmation probability to 84.7%. However, a significant difference was noted between shorter and longer disease durations (PPV < 5 years 70.6%, > 5 years 89.1%). Including a smell test (UPSIT, normal values 34–40, study cutoff < 22 points) within the first 2 years of consultations significantly increased positive predictive value from 22% to 83.3% for patients with possible PD and to 89.7% for patients with probable PD. Thus, particularly in early PD stages, hyposmia enhances positive predictive value and diagnostic certainty [32, 45].

#### Recommendation (new in German guideline, 2023)

Olfactory testing, e.g., with the Sniffin’ Sticks test, increases the positive predictive value of a clinical diagnosis of PD to ≥ 80% after excluding common alternative causes of hyposmia, thereby enhancing the diagnostic certainty of PD. Due to its low invasiveness, it is recommended as an adjunctive diagnostic tool. However, the presence of normosmia does not exclude PD.

*Consensus strength:* 97.3%, strong consensus.

### Question 9: what is the predictive value of standardized polysomnography for detecting isolated REM sleep behavior disorder, as compared to the clinical follow-up for the diagnosis of PD?

#### Background

REM sleep behavior disorder (RBD) is a non-motor symptom of PD that can increase diagnostic certainty. It is characterized by vivid acting-out of dreams, sometimes with vocalizations, and is identified in sleep studies by the absence of REM atonia. Diagnosis of RBD involves history-taking by patients and spouses, and polysomnography, with confirmation required according to ICSD-3 criteria. RBD symptoms can be assessed via questionnaires to diagnose possible RBD, but these have limited diagnostic reliability due to many patients being unaware of their symptoms, and various mimics such as other parasomnias, periodic limb movements (PLMS), and severe sleep-related breathing disorders that can mimic or provoke RBD symptoms. The gold standard for diagnosis remains polysomnography in a sleep laboratory, which captures behavioral manifestations such as punching, kicking, and shouting, and definitely diagnoses RBD by demonstrating incomplete or absent REM atonia while excluding RBD mimics.

#### Results

The sensitivity and particularly the specificity of diagnosing REM sleep behavior disorder (RBD) are low when using questionnaires alone. The highest sensitivity and specificity are achieved through a combination of history by patients and spouses, and polysomnography [46–48]. However, clinical and polysomnographic criteria may only be fully met over time as symptoms intensify. In disease progression, patients with clinically isolated RBD (iRBD) and hyposmia appear to experience faster progression, meeting criteria for manifest PD or other neurodegenerative diseases (conversion), despite a significant portion continuing to exhibit normosmia even after a PD (up to 44%). In addition, in PD patients, the presence of hyposmia and RBD is associated with a worse prognosis concerning increased motor impairments and cognitive deficits. The likelihood ratio (LR) for polysomnographically confirmed RBD is 130, as outlined in initial prodromal criteria. RBD was found in 42.3% of Parkinson’s patients in a meta-analysis of 28 studies, reflecting its high LR in the prodromal phase due to its specificity. However, since only a subset of PD patients develops RBD during the course of the disease, its high predictive value is contingent upon the presence of this symptom. There is currently no literature comparing the predictive value of iRBD against clinical follow-up.

#### Recommendation (new in German guideline, 2023)

Testing for the presence of RBD is helpful for the diagnosis of PD. The diagnosis of probable RBD should be made based on history by patients and spouses rather than relying solely on questionnaires. A definitive diagnosis of RBD should involve a polysomnographic examination in a sleep laboratory.

*Consensus strength*: 100%, strong consensus.

### Question 10a: what is the value of a validated smell test in distinguishing PD from atypical Parkinson syndromes like PSP or MSA?

#### Background

Impairments in the sense of smell (hyposmia/anosmia) are among the most common non-motor symptoms in PD and often occur very early in the course of the disease. At the same time, especially early in the disease course, it can be challenging to differentiate atypical Parkinson syndromes from PD, highlighting the importance of clinical and diagnostic markers in distinguishing between these different disease entities.

#### Results

Longitudinal studies in small patient groups with REM sleep behavior disorder indicate that patients who developed MSA typically exhibited normosmia compared to those who developed PD [38, 49]. In addition, a small cross-sectional study comparing PSP patients to PD patients found normal olfactory function in PSP patients [50]. Other smaller cross-sectional studies in MSA and PSP patients either found no differences compared to controls or, at most, significantly lower hyposmia than in PD patients. Using the Identification subtest of the Sniffin’ Sticks test, a positive predictive value of 85.7% and a negative predictive value of 78.5% were demonstrated in distinguishing PD from MSA or PSP, which was comparable to the total test (SDI Score) but required significantly less time for administration. Using the best cutoff with high specificity enabled reliable differentiation between PD and atypical Parkinson syndromes, with better discrimination observed between PD and MSA. A meta-analysis involving over 1000 included patients confirmed that olfactory impairments in PD are significantly more pronounced compared to PSP, with no significant differences found compared to patients with iRBD [51].

#### Recommendation (new in German guideline, 2023)

Testing olfactory function, for example using the Sniffin’ Sticks test, may be considered as supportive examination, after excluding symptomatic causes of hyposmia. In cases of pronounced hyposmia or anosmia, this can support the diagnosis of PD as opposed to MSA and PSP.

*Consensus strength*: 97.2%, strong consensus.

### Question 10b: what is the value of standardized polysomnographic RBD diagnosis in distinguishing PD from atypical Parkinson syndromes like PSP or MSA?

#### Background

Clinical isolated REM sleep behavior disorder (iRBD) is characterized by vivid, potentially harmful acting-out of dreams, sometimes with vocalizations, and observable lack of REM atonia in sleep lab settings. RBD symptoms can be assessed through questionnaires (possible RBD), but many patients are unaware of their symptoms, often requiring informant input to suggest RBD. The gold standard for diagnosis is polysomnographic examination in sleep laboratories, where, in addition to behavioral abnormalities such as punching, kicking, and yelling, the diagnosis is confirmed by the presence of incomplete or absent REM atonia.

#### Results

Patients with polysomnographically confirmed clinically isolated REM sleep behavior disorder (iRBD) are highly likely (> 90%) to develop a neurodegenerative disease over time, with 98% developing alpha-synucleinopathies such as PD/DLB or MSA [52–54]. Thus, RBD is considered a prodromal stage not only of PD but also of alpha-synucleinopathies in general, with up to 8% of patients developing MSA [52]. However, RBD also occurs in atypical Parkinson syndromes like PSP, vascular lesions in the brainstem, and other neurodegenerative diseases including Alzheimer’s dementia [55–58]. Nonetheless, RBD is much less common in other neurodegenerative diseases compared to alpha-synucleinopathies and may occur in conjunction with Lewy body and tau pathology [55]. Reported frequencies vary significantly due to small sample sizes. In PSP, RBD has been observed in 20–50% of patients, while in MSA, one study found absent REM atonia in 90% of patients via polysomnography, though smaller studies report frequencies around 20% [57]. Polysomnographic changes in RBD do not fundamentally differ between patients with PD and MSA but may vary in severity depending on the cohort, often due to small sample sizes [59, 60].

#### Recommendation (new in German guideline, 2023)

Polysomnography for confirming RBD can be considered. If RBD is detected, the presence of an alpha-synucleinopathy (PD and MSA) is more likely than a tauopathy. However, the absence of RBD does not exclude an alpha-synucleinopathy, and differentiation between different alpha-synucleinopathies cannot be made.

*Consensus strength*: 100%, strong consensus.

### Question 11: how effective is prognosis estimation in PD considering non-motor symptoms (RBD, hyposmia, constipation, depression, orthostasis) compared to purely motor criteria?

#### Background

Diagnosing a chronic (neurodegenerative) disease like PD often causes uncertainty and anxieties about the future for patients and their families, who seek reliable information regarding prognosis and treatment options. These decisions can influence significant life choices such as living arrangements and therapeutic limitations. Furthermore, predicting disease progression is crucial for healthcare providers in selecting both pharmacological and supportive therapies. It is also of scientific interest in defining outcome and prognostic parameters. PD is a heterogeneous disease with various subtypes, traditionally classified based on purely motor criteria (including akinetic-rigid and tremor-dominant types), but increasingly incorporating non-motor symptoms.

#### Results

In a 2007 meta-analysis including 27 studies, poorer prognosis and faster progression of motor deficits were associated with pre-existing limitations in activities of daily living, cognitive impairments, and depressive symptoms [61]. The prevalence RBD in PD was reported as 46% in a 2023 meta-analysis. The presence of RBD was also linked to faster progression of motor symptoms and fluctuations, older age, lower education level, higher doses of dopaminergic therapy (levodopa equivalent dose), longer disease duration, as well as more pronounced autonomic and neuropsychiatric symptoms such as cognitive abnormalities and hallucinations [62]. Another meta-analysis on the influence of depression on PD progression included 129 studies with a total of 38,304 participants from 28 countries. The overall prevalence of depression in PD patients was estimated at 38%. Patients with depression exhibited earlier disease onset, lower educational attainment, worse cognitive performance, and more severe motor symptoms, with associations noted with gait disturbances, particularly freezing. In addition, the occurrence of depression was associated with female gender and other non-motor symptoms such as anxiety, apathy, and fatigue symptoms. A retrospective cohort study published in 2019 analyzed longitudinal data collected between 2009 and 2017 from 111 autopsied PD patients [63]. Based on cluster analyses of the PPMI cohort, patients were classified into predominant mild-motor, intermediate, or diffuse malignant subtypes based on the severity of their motor, cognitive, autonomic, and depressive symptoms, as well as the presence or absence of RBD at diagnosis [64]. Each subtype’s time to reach specific disease milestones such as recurrent falls, wheelchair dependency, dementia, institutionalization, and death was calculated. Patients with the diffuse malignant subtype reached these milestones significantly earlier than other subtypes, with age identified as the sole relevant cofactor. This subtype primarily consisted of older men with poor response to levodopa and was often misclassified as atypical Parkinsonism. Despite earlier milestone attainment, all subtypes experienced similar degrees of impairment at death. Other smaller cohort studies have also found associations in Parkinson’s patients between RBD, hyposmia, poorer motor outcomes and cognitive deficits [42, 43].

#### Recommendation (new in German guideline, 2023)

The severity of non-motor symptoms should be considered for evaluating the prognosis already at the time diagnosis in PD.

*Consensus strength:* 100%, strong consensus.

## Imaging diagnostics

The chapter on imaging diagnostics addresses the following questions:

*Question 12:* how effective is cranial CT (cCT) compared to long-term clinical follow-up for the differential diagnosis of Parkinson’s disease (PD) versus secondary Parkinsonian syndromes?

*Question 13:* how effective is magnetic resonance imaging (MRI), considering various data acquisition techniques (MR sequences) and post-processing strategies, compared to long-term clinical follow-up for diagnosing Parkinson’s disease (PD)?

*Question 14:* how effective is brain parenchymal sonography in differentiating Parkinson’s disease (PD) from atypical and secondary Parkinsonian syndromes?

*Question 15:* how effective is brain parenchymal sonography in differentiating Parkinson’s disease (PD) from essential tremor?

*Question 16:* how effective is brain parenchymal sonography compared to clinical follow-up for diagnosing Parkinson’s disease (PD) in individuals with typical early symptoms*?

*Question 17:* how effectively does FDG-PET differentiate Parkinson’s disease (PD) from other diagnoses (MSA/PSP/CBD) compared to long-term clinical follow-up?

*Question 18:* how effective is FDG-PET compared to long-term clinical follow-up in predicting the occurrence of dementia in Parkinson’s disease (PD)?

*Question 19:* how effective is presynaptic single-photon emission computed tomography of the striatum (DAT-SPECT) compared to long-term clinical follow-up for diagnosing a neurodegenerative Parkinsonian syndrome?

*Question 20:* how effectively does postsynaptic single-photon emission computed tomography of the striatum (IBZM-SPECT) differentiate Parkinson’s DISEASE (PD) from other diagnoses (MSA/PSP/CBD) compared to long-term clinical follow-up?

*Question 21:* how effective is cardiac MIBG scintigraphy or single-photon emission computed tomography in differentiating Parkinson’s disease (PD) from multiple system atrophy (MSA) compared to long-term clinical follow-up?

*Question 22:* how effective is cardiac MIBG scintigraphy or single-photon emission computed tomography in differentiating Parkinson’s disease (PD) from 4-repeat tauopathies (PSP and CBD) compared to long-term clinical follow-up?

### Background

Especially in the early motor stages of the disease, distinguishing PD from atypical and secondary Parkinson syndromes based solely on clinical criteria can be challenging [9]. Therefore, there is a need for additional diagnostic methods to increase diagnostic accuracy. Cranial MRI provides valuable diagnostic assistance in the differential diagnosis of Parkinson syndromes, including the exclusion of symptomatic causes and differentiation from other neurodegenerative Parkinson syndromes (given the high positive predictive value of corresponding MRI signs for other neurodegenerative Parkinson syndromes). At the onset of symptoms, distinguishing PD) from other neurodegenerative disorders with Parkinsonian features, particularly MSA, PSP, and CBD, presents a clinical challenge [13]. FDG-PET of the brain is an established routine procedure that can capture and diagnostically utilize the impact of the disease on brain metabolism [65, 66].

### Recommendation (new in German guideline, 2023), summary of questions 12–22

Cranial MRI (cMRI) should be conducted early in the disease course to aid in the differential diagnosis of Parkinson syndromes. For evaluating exclusion criteria for PD, cMRI scans with standardized sequences, including T1-weighted and T2-weighted (preferably high-resolution 3D). In addition, iron-sensitive/susceptibility-weighted and diffusion-weighted sequences may be included.

*Consensus strength*: 97%, strong consensus.

Transcranial brain parenchymal sonography (TCS) performed by a qualified examiner can be useful in differentiating PD from atypical and secondary Parkinsonian syndromes. TCS should assess the substantia nigra, nucleus lentiformis, and the third ventricle.

*Consensus strength:* 97.4%, strong consensus.

FDG-PET may be considered if clinical signs strongly suggest an atypical Parkinson syndrome and the results will impact clinical decisions, such as diagnosis, prognosis, or therapy.

*Consensus strength:* 84%, consensus.

FDG-PET may also be used to evaluate the risk of dementia in PD, provided the findings have clinical implications.

*Consensus strength*: 97%, strong consensus.

Dopamine transporter SPECT (DAT-SPECT) may be performed early in the disease course to detect nigrostriatal deficits in cases where the diagnosis of Parkinson or tremor syndromes is unclear, if the results will influence clinical management.

*Consensus strength*: 82.8%, consensus.

Cardiac MIBG scintigraphy or SPECT can be considered to distinguish PD from MSA if FDG-PET is not available.

*Consensus strength*: 100%, strong consensus.

## Genetic diagnostics

The chapter on genetic diagnostics addresses the following questions:

*Question 27a:* in which patients with PD) is there a well-founded suspicion of a monogenic cause?

*Question 27b:* in which patients with Parkinson’s disease (PD) should genetic counseling and genetic testing be offered?

*Question 28:* what phenotypic and other characteristics in the patient groups defined in the questions above lead to the recommendation of which examination?

*Question 29:* which examination has the highest success rate and the lowest rate of false-negative/false-positive results? Which examination is cost-effective?

*Question 30:* how effective is genetic testing in the groups defined in question 1 for an etiologically accurate diagnosis?

*Question 31:* how effective is a genetically confirmed diagnosis of hereditary Parkinson’s disease for predicting prognosis regarding survival, quality of life, and cognitive decline?

*Question 32:* how effective is a genetically confirmed diagnosis of hereditary Parkinson’s syndrome for treatment decision-making?

*Question 33:* is there a group of Parkinson’s patients in whom complex genetic factors should be considered?

### Background

Hereditary Parkinson syndromes, caused by pathogenic variants in a single gene, are rare. Genetic diagnostics can confirm these diagnoses, which is important for making informed statements about prognosis, optimal therapy, and disease risk for family members. A suspected monogenic cause is primarily based on the age of onset and family history. Hereditary Parkinson syndromes are named using the term “PARK” followed by the gene abbreviation carrying the pathogenic variant, e.g., PARK-LRRK2 for a variant in the LRRK2.

Monogenic diseases are studied through numerous small family and case studies. These studies are heterogeneous, leading to significant variability in symptom frequency, disease progression, and other parameters. Publication bias towards unusual and extreme phenotypes also occurs over time. As a result, a standardized classification into three evidence classes, typically used in these guidelines, is not applicable. The MDSGene database (as of 01.05.2023, https://www.mdsgene.org/) aims to collect and analyze all publications on monogenic forms of movement disorders [67, 68]. Data regarding age of onset and initial symptoms were obtained from MDSGene. The number of cases per gene is generally low, especially for DJ1, VPS35, SNCA, and PINK1, leading to variability in the data. Initial symptom data is heterogeneous, with up to 60% of cases lacking specific symptom information. However, age of onset data is relatively complete (< 10% missing for most genes except DJ1 at 19%).

### Recommendation (new in German guideline, 2023), summary of questions 27a–33

Mutations in four genes are known to cause autosomal-dominant hereditary PD (*LRRK2*, *SNCA*, *VPS35*, and *CHCHD2*), while three (*PRKN*, *PINK1*, and *DJ1*) cause forms of the syndrome through biallelic pathogenic variants, meaning both gene copies must carry the variant to cause the disease (Table 3) [67, 68]. Mutations in genes causing atypical, often juvenile hereditary Parkinson syndromes (e.g., *ATP13A2*, *DNAJC6*, *FBXO7*, *SLC6A3*, and *SYNJ1*) are not covered in this guideline, as these syndromes are very rare. Recently, *CHCHD2* was also recognized as a cause of hereditary PD by the International Parkinson and Movement Disorders Society’s Task Force for Nomenclature and Classification of Genetic Movement Disorders. Data for *CHCHD2* includes 24 publications [69, 70].

**Table 3:** Hereditary PD, causal genes, mode of inheritance, age of onset, number of patients, as analyzed in MDSGene

Form	Gene	Mode of inheritance	Age of onset	Number of patients in MDSGene (www.mdsgene.org)
PARK-LRRK2	*LRRK2*	Autosomal dominant with reduced penetrance*	Median: 57; 25th/75th percentile: 47/65 (range: 24–91 years)	723
PARK-SNCA	*SNCA*	Autosomal dominant with reduced penetrance*	Median: 46; 25th/75th percentile: 36/54 (range: 19–77 years)	146
PARK-VPS35	*VPS35*	Autosomal dominant with reduced penetrance*	Median: 52; 25th/75th percentile: 45/61 (range: 26–75 years)	68
PARK-DJ1	*DJ1* (PARK7)	Autosomal recessive with high penetrance	Median: 27; 25th/75th percentile: 22/34 (range: 14–40 years)	35
PARK-PRKN	*PRKN*	Autosomal recessive with high penetrance	Median: 31; 25th/75th percentile: 23/38 (range: 3–81 years)	1487
PARK-PINK1	*PINK1*	Autosomal recessive with high penetrance	Median: 32; 25th/75th percentile: 25/40 (range: 9–67 years)	180

*With the exception of *SNCA* triplication and pathogenic single nucleotide variants in SNCA, which are fully penetrant

Diagnostic genetic testing should be offered upon patient request if either two first-degree relatives or one first-degree and one second-degree relative were diagnosed with PD, or if the disease manifests before age 50.

*Consensus strength*: 96.4%, strong consensus.

For PD patients with onset age over 50, at least the *LRRK2*, *SNCA*, and *VPS35* genes should be examined. Besides sequencing, the techniques used should also detect deletions and duplications.

*Consensus strength*: 100%, strong consensus.

For PD patients with disease manifestation before age 50 who request genetic testing, the *PRKN*, *PINK1*, *DJ1*, *LRRK2*, *SNCA*, and *VPS35* genes should be examined. If multiple family members are affected, testing should preferably start with the patient with the youngest age of onset, using appropriate techniques (sequencing, detection of deletions/duplications).

*Consensus strength*: 100%, strong consensus.

If suspicion of a genetic cause of PD persists despite negative findings in the aforementioned tests, a neurologist specializing in neurogenetics or a geneticist should be consulted if the patient wishes to pursue further diagnostic procedures.

*Consensus strength*: 100%, strong consensus.

Investigations to assess polygenic risk should not be routinely performed in clinical care.

*Consensus strength*: 96.2%, strong consensus.

Genetic variant testing for the *GBA1* gene should also not be routinely performed. However, in cases of isolated PD, disease manifestation before age 50, or in patients with rapid motor progression or fast cognitive deterioration, examination of the *GBA1* gene may be considered if the patient requests it after giving informed consent.

*Consensus strength*: 96.2%, strong consensus.

Diagnosing hereditary PD does not allow for reliable prediction of survival, quality of life, or cognitive impairment for individual patients, and there is no specific approved pharmacological therapy for either monogenic PD or genetically complex PD to date. Deep brain stimulation is possible in monogenic PD, with the same inclusion and exclusion criteria as in genetically complex, sporadic PD.

*Consensus strength*: 100%, strong consensus.

*Question 34:* how effective is the analysis of molecular biomarkers (in CSF, blood, skin, and stool) compared to long-term clinical follow-ups in diagnosing Parkinson’s disease (PD)?

### Background

To date, few biomarkers in biological fluids have been routinely used in Parkinson’s disease.

### Recommendation (new in German guideline, 2023), question 34

Neurofilament light chain (NfL), a marker of axonal damage, is elevated in cerebrospinal fluid and blood across various neurological diseases, but is not specific to a particular disease [71]. Studies show slightly higher average NfL levels in PD patients compared to healthy controls, with significantly higher levels in atypical Parkinsonian syndromes such as PSP and MSA [72]. NfL quantification is available for clinical use in both blood and CSF.

Alzheimer’s biomarkers, such as β-amyloid 1–42 and total tau protein in CSF, were found to be decreased or normal in PD in a prospective study. A low β-amyloid 1–42 level in CSF may indicate a higher risk of cognitive decline in PD patients [73, 74].

Recently, methods have emerged to detect misfolded α-synuclein in CSF with high sensitivity using seed amplification assays (SAA). These assays, similar to prion assays, amplify the pathological aSyn protein and can be measured semi-quantitatively. A large meta-analysis showed consistent sensitivities and specificities above 90% across various labslaboratories [75]. These high values were also replicated in a large international multicenter cohort. SAA could potentially detect PD up to 10 years before clinical onset, making it a promising early marker [76]. Studies are ongoing to establish this assay in peripheral fluids and tissues, such as saliva, skin biopsies, olfactory epithelium, or blood. While showing great potential as a diagnostic biomarker for α-synuclein pathology, SAA is not yet approved for clinical routine in Germany [77].

### Recommendation (new in German guideline, 2023)

NfL (from CSF or serum) is not suitable as a diagnostic biomarker for PD due to a lack of specificity but may help differentiate PD from atypical Parkinsonian syndromes.

*Consensus strength*: 100%, strong consensus.

## Discussion

This guideline underscores the importance of a structured and comprehensive approach to PD diagnosis. By establishing key PICO questions, the steering committee ensured a focused and systematic literature review, which was critical in forming evidence-based recommendations. The collaborative effort of chapter authors and the iterative process of refining these recommendations through consensus voting illustrate the guideline’s robust methodological framework.

The evolving understanding of PD and its genetic underpinnings necessitates a reevaluation of traditional terminologies and diagnostic criteria. Historically, the terms “Parkinson’s disease” (PD) and “idiopathic Parkinson’s syndrome” (IPS) were used interchangeably. However, recent advances in genetic research reveal that a substantial number of PD cases are linked to genetic variants, challenging the idiopathic nature of the disease. Consequently, the Parkinson’s Guideline Group of the German Society for Neurology now advocates for the broader term PD to encompass both idiopathic and hereditary forms.

The distinction between hereditary and sporadic PD is particularly noteworthy. Hereditary PD, driven by specific pathogenic genetic variants, often follows clear Mendelian inheritance patterns, whereas sporadic PD involves a complex interplay of numerous genetic factors with each variant contributing modestly to disease risk. This polygenic nature of sporadic PD is a frontier of ongoing research, promising to enhance our understanding of the disease’s genetic complexity and pave the way for more personalized treatment strategies. Although there are many interesting, promising approaches for diagnostic biomarkers in Parkinson’s disease, no clinical use is currently recommended due to a lack of specificity and quality assessment of the biomarker tests.

Currently, the MDS criteria for the clinical and prodromal diagnosis of PD are valid and intensively applied in clinical practice [9, 13, 14].

The new recommendations published 2023 reflect a shift towards more nuanced diagnostic criteria and long-term disease management. Long-term clinical follow-up emerges as a superior strategy for accurate diagnosis, highlighting the dynamic nature of PD and the necessity for ongoing patient reassessment.

Moreover, the guidelines emphasize the importance of considering non-motor symptoms, olfactory testing, and RBD in the diagnostic process. These aspects not only enhance diagnostic certainty but also provide valuable insights into the disease’s progression and prognosis. The inclusion of olfactory tests and polysomnography as supportive diagnostic tools represents a move towards more comprehensive diagnostic practices.

The strong consensus on most recommendations reflects a high degree of agreement among experts, reinforcing the credibility and reliability of the guidelines. Recommendations such as monitoring levodopa-responsiveness, but not recommending routine use of acute levodopa and apomorphine tests, while considering their diagnostic value in specific contexts, exemplify a differentiated approach to PD diagnosis. The focus on long-term follow-up to monitor development of PD-supporting motor complications or possible appearance of red flags arguing in favor of atypical Parkinson syndromes, and the recommendation of the MDS criteria for diagnosis of prodromal or established PD underscore the guidelines’ commitment to improving diagnostic accuracy as novel evidence emerges.

In summary, the 2023 German guidelines for Parkinson’s disease represent a significant advancement in the field, integrating recent genetic insights and emphasizing long-term, patient-centered approaches to diagnosis and management. As our understanding of PD continues to evolve, these guidelines provide a crucial framework for clinicians, ensuring that diagnostic and therapeutic strategies are both evidence-based and adaptable to the complexities of the disease.

## Data Availability

The data presented in this article have been extracted from the guideline ‘Parkinson’s Disease’ by the German Society of Neurology: https://dgn.org/leitlinie/parkinson-krankheit.
